# Vonoprazan versus proton pump inhibitors for the management of gastroesophageal reflux disease: A protocol for a systematic review with meta-analysis

**DOI:** 10.1097/MD.0000000000012574

**Published:** 2018-09-28

**Authors:** Hyun Kang, Beom Jin Kim, Geunjoo Choi, Jae Gyu Kim

**Affiliations:** a Department of Anesthesiology and Pain Medicine; b Department of Internal Medicine, Chung-Ang University Hospital, Chung-Ang University College of Medicine, Seoul, Korea.

**Keywords:** gastroesophageal reflux disease, meta-analysis, proton pump inhibitors, systematic review, vonoprazan

## Abstract

**Background and Aim:**

Vonoprazan, a novel potassium-competitive acid blocking agent, is used in the management of gastroesophageal reflux disease (GERD). We aim to perform a systematic review and meta-analysis for the comparison of the effects of vonoprazan and proton pump inhibitors (PPIs) in GERD in randomized controlled trials (RCTs).

**Methods:**

A systematic and comprehensive search will be performed using MEDLINE, EMBASE, Cochrane Central Register of Controlled Trials (CENTRAL), Google Scholar, and clinical trial registries, for studies published up to September 2018. Only randomized clinical trials will be included. Primary outcomes of symptoms and esophageal erosion improvement in the intention-to-treat analysis, and secondary outcomes of symptoms and esophageal erosion improvement rate in the per protocol analysis, the comparative efficacy in terms of healing rate of esophageal erosion on endoscopy, the comparative efficacy in terms of improvement of esophageal impedance-pH study, adverse events, long-term safety, and the comparative efficacy in terms of CYP2C19 metabolite levels will be studied. The quality of included studies will be assessed using the modified risk of bias tool. Heterogeneity of estimates across studies as well as publication bias will be assessed. This systematic review and meta-analysis will be performed according to the protocol recommended by the Cochrane Collaboration and reported according to the preferred reporting items for systematic reviews and meta-analysis (PRISMA) guidelines. All statistical analyses will be conducted using Stata SE version 15.0.

**Results:**

The results of this systematic review and meta-analysis will be published in a peer-reviewed journal.

**Conclusion:**

To our knowledge, this systematic review and meta-analysis will be the first to evaluate existing research comparing Vonoprazan and PPIs in GERD. Our study will provide information about the effect of vonoprazan and PPIs in GERD in RCTs. The review will benefit patients, healthcare providers, and policymakers.

**Strengths and limitations**

- This systematic review and meta-analysis will provide a comprehensive and objective comparison of the effect of vonoprazan and proton pump inhibitors (PPIs) in gastroesophageal reflux disease (GERD) in randomized controlled trials
- The study will provide useful and novel information for patients, healthcare providers, and policymakers.
- The study will assess the methodological and reporting qualities of included studies using modified risk of bias tool.
- Our results may be limited by heterogeneity due to differences in type of study, the different kinds of PPIs investigated, and diverse dosage regimens of PPI administration.

## 1 Introduction

Gastroesophageal reflux disease (GERD) is a troublesome condition that causes symptoms such as heartburn and acid regurgitation by reflux of stomach contents.^[1]^ GERD is prevalent in Western countries, and recently it has been dramatically increasing in many Asian countries.^[2–4]^ GERD includes erosive esophagitis (EE) and non-erosive reflux disease (NERD) diagnosed by esophagogastroduodenoscopy, but the severity of symptoms is not necessarily proportional to the degree of mucosal injury.^[5,6]^

Generally, proton pump inhibitors (PPIs) have been the mainstay for the management of GERD.^[7]^ Indeed, PPIs have been beneficial in patients with both EE and NERD,^[8,9]^ and patients with EE showed 20% greater improvement of GERD symptoms than patients with NERD.^[10]^ Although PPIs are widely used in clinical practice, the standard dose of PPI does not always induce sufficient gastric acid suppression in all patients because of their pharmacological limitations.^[11,12]^ In fact, 10% to 20% of patients with severe EE (Los Angeles classification C and D) do not heal despite 8 weeks of continuous double-dose PPI therapy.^[13]^ Moreover, it has been well documented that achieving complete symptomatic relief with PPI is more difficult than simply healing mucosal breaks, resulting in dissatisfaction of current therapy in about one-third of patients with GERD.^[14]^

Recently, a novel potassium-competitive acid blocking (P-CAB) agent called vonoprazan (TAKECAB; Takeda Pharmaceutical Co. Ltd., Tokyo, Japan), has been developed that is stronger, faster, and exhibits longer-lasting acid suppression than conventional PPIs.^[15,16]^ The acid-inhibitory effect of vonoprazan has been reported to be more potent than that of PPIs, with greater impact against acid-related diseases such as GERD, *Helicobacter pylori* infection, gastric and duodenal ulcers, and prevention of recurrence in nonsteroidal anti-inflammatory-drug or low-dose-aspirin ulcer.^[17,18]^

Vonoprazan may have an efficacy comparable to or better than that of PPIs in the treatment of GERD. There is a growing number of reports comparing the effectiveness of vonoprazan with that of PPIs in treating GERD.^[2,19–21]^ However, the findings have been variable and reported outcomes are conflicting. Furthermore, no previous systematic review and meta-analysis have been published regarding this issue. Therefore, we developed the protocol for a systematic review and meta-analysis to assess and compare the effects of vonoprazan and PPIs in the treatment of GERD in randomized clinical trials (RCT).

## 2 Methods

We developed the protocol for our systematic review and meta-analysis according to the preferred reporting items for systematic reviews and meta-analysis protocols (PRISMA-P) statement,^[22]^ and registered it in the international prospective register of systematic reviews PROSPERO network (registration number: CRD42018091655; www.crd.york.ac.uk/PROSPERO).

This systematic review and meta-analysis comparing the effects of vonoprazan and PPIs for the management of GERD will be performed in accordance with the protocol recommended by the Cochrane Collaboration^[23]^ and will be reported according to the preferred reporting items for systematic reviews and meta-analysis (PRISMA) guidelines.^[24]^

### 2.1 Ethical issues

This systematic review does not require ethical approval or informed consent because there will be no direct contact with individual patients, and only previously published data will be included in the review.

### 2.2 Inclusion and exclusion criteria

#### 2.2.1 Types of studies

Only randomized controlled studies (parallel design and cross-over design) will be eligible for inclusion. Studies in any language published until September 2018 will be included. Cohort studies, review articles, case-control studies, case reports, case series, letters to the editor, commentaries, proceedings, laboratory science studies, and any other non-relevant studies will be excluded from analysis.

#### 2.2.2 Population

Inclusion criteria for study populations will be all patients with GERD, including EE and NERD. No restrictions will be applied in terms of age, sex or ethnicity.

#### 2.2.3 Intervention and comparison

Interventions to be examined will include medication with potassium-competitive acid blocker including vonoprazan and comparator will be medication with PPIs including lansoprazole, esomeprazole, rabeprazole, etc.

#### 2.2.4 Outcome measures

The primary outcomes are symptoms and improvement rate of esophageal erosion in the intention-to-treat analysis (O), and the secondary outcomes are symptoms and improvement rate of esophageal erosion in the per-protocol analysis, the comparative efficacy in terms of healing rate of esophageal erosion on endoscopy, the comparative efficacy in terms of improvement of esophageal impedance-pH study, adverse events, long-term safety and the comparative efficacy in terms of CYP2C19 metabolite levels.

### 2.3 Data sources

A search will be performed in MEDLINE, EMBASE, Cochrane Central Register of Controlled Trials (CENTRAL) and Google Scholar, for studies published up to September 2018. We will also search registered trials described in clinical trial registries, which are listed in the Appendix.

The search terms included vonoprazan, Takecab, P-CAB, TAK-438, potassium-competitive, GERD, EE, NERD and GERD.

Two authors will screen titles and abstracts of the retrieved articles. Reference lists will be imported to Endnote 8.1 (Thompson Reuters, CA), and duplicate articles will be removed. Additional relevant articles will be identified by scanning reference lists of articles obtained from the original search.

### 2.4 Study selection

The titles and abstracts identified through the search strategy described above will be scanned independently by 2 authors. To minimize data duplication as a result of multiple reporting, papers from the same author and organ will be compared. For reports determined to be eligible based on the title or abstract, the full paper will be retrieved. Potentially relevant studies chosen by at least 1 author will be retrieved and evaluated in full-text versions. Articles meeting the inclusion criteria will be assessed separately by 2 authors, and any discrepancies will be resolved through discussion. In cases where agreement cannot be reached, the dispute will be resolved with the help of a third investigator. A flow diagram for the search and selection process will be developed following PRISMA guidelines.

### 2.5 Data extraction

Using a standardized extraction form, the following data will be extracted independently by 2 authors: study name (along with the name of the first author and year of publication), country where the study was conducted, study design, country, study period, publication language, number of patients, types and doses of intervention and comparator medication, symptom and esophageal erosion improvement rate, healing rate of esophageal erosion on endoscopy, improvement of esophageal impedance-pH study, adverse events, long-term safety and CYP2C19 metabolite levels.

If information is missing, an attempt will be made to contact the study authors to obtain the relevant information. When unsuccessful, missing information will be calculated if possible from the relevant data within the study.

The reference list will be divided into 2 halves. Two authors will complete data extraction, 1 for each half of the reference list. Data extraction forms will be cross-checked to verify accuracy and consistency of the extracted data.

### 2.6 Study quality assessment

The quality of the studies will be independently assessed by 2 authors using the “risk of bias” tool for RCTs according to the Review Manager (version 5.3, The Cochrane Collaboration, Oxford, UK).^[23]^ Quality will be evaluated using the following potential sources of bias: sequence generation, allocation concealment, blinding of participants, procedure performer (anesthesiologist and intervention implementer), outcome assessor, incomplete data, and selective reporting. The methodology for each study was graded as “high”, “low,”, or “unclear”, which reflected a high risk of bias, low risk of bias, and uncertain bias, respectively.

### 2.7 Statistical analysis

Ad-hoc tables will be designed to summarize data from the included studies and show their key characteristics and any important questions related to the aim of this review. After data have been extracted, reviewers will determine whether a meta-analysis is possible.

### 2.8 Statistical analysis

We will compute the pooled relative risk (RR) with 95% confidence intervals (CI) for dichotomous data, and standardized mean difference (SMD) or mean difference (MD) with 95% CI for continuous data.

Between-study heterogeneity will be assessed using the Cochran Q and Higgins I^2^ statistics. A *P* value of <.10 for the Chi^2^ statistic or an I^2^ greater than 50% will be considered as showing considerable heterogeneity, and data will be analyzed using the Mantel–Haenszel random-effect model. Otherwise, we will apply the Mantel–Haenszel fixed-effect model.^[23,25]^

If the number of studies with substantial heterogeneity is less than 10, the *t* statistic (Hartung-Knapp-Sidik-Jonkman method) will be used instead of the *Z* test in all random effects analysis to decrease the error rate.^[26]^ We will conduct sensitivity analyses to evaluate the influence of a single study on the overall estimate by excluding 1 study at a time in case of substantial heterogeneity. We will calculate the number needed to treat based on absolute risk reduction as an estimate of the overall clinical impact of the intervention.^[27]^

Publication bias will be assessed by using Begg funnel plot and Egger test. Begg funnel plots are scattered plots of the log ORs of individual studies on the *x*-axis against 1/standard error (SE) of each study on the *y*-axis. Egger test is a test for linear regression of the normalized effect estimate (log OR/SE) against its precision (1/SE).^[28]^ An asymmetrical funnel plot or a *P* value of < .1 from Egger's test will be considered to indicate the presence of publication bias. If publication bias is detected, trim and fill analyses will be performed.^[29]^ If fewer than 10 studies are included, publication bias will not be assessed.^[25]^ If data are reported as a median (*P*_25_ –*P*_75_), median (range) or mean (SE of mean), we will calculate the mean and standard deviation from these values.^[30]^ We will perform all analyses using Review Manager software (version 5.3, The Cochrane Collaboration, Oxford, UK) and Stata SE version 15.0 (StataCorp, College Station, TX).

### 2.9 Evidence synthesis

The evidence grade will be determined using the guidelines of the Grading of Recommendations, Assessment, Development, and Evaluation (GRADE) system which uses sequential assessment of the evidence quality that is followed by an assessment of the risk-benefit balance and a subsequent judgment on the strength of the recommendations.^[31]^

## 3 Discussion

GERD is a disease comprising symptoms and complications related to the reflux of gastric contents into the esophagus.^[32]^ Although PPIs are used as a first-line treatment of GERD, epidemiologic estimates show that approximately 20% to 40% of patients with GERD are poor responders to PPI therapy.^[33,34]^ Furthermore, conventional PPIs cannot satisfy the expected therapeutic effect in terms of acid suppression.

As a novel acid suppressant, the effect of vonoprazan on the treatment of GERD has been evaluated. Various clinical studies have reported that vonoprazan is comparable or superior to PPIs for the treatment of GERD including EE and NERD.^[19,20]^ However, it remains unclear whether vonoprazan is superior to PPIs for the treatment of GERD. Therefore, a meta-analysis and systematic review in this field may lead a more accurate conclusion.

We designed this systematic review and meta-analysis for the purpose of comparing efficacy of vonoprazan with PPIs for the management of GERD. This study will merge all the current evidence and provide suggestions for clinical practice. To the best of our knowledge, this study will provide the first evidence that vonoprazan may be considered an effective treatment for GERD. Meanwhile, the result of this meta-analysis will add knowledge in the comparative effectiveness of current pharmacological treatment, which helps clinicians make the best decisions on their first-choice drug for GERD.

## Author contributions

**Conceptualization:** Beom Jin Kim.

**Data curation:** Geunjoo Choi.

**Formal analysis:** Hyun Kang, Beom Jin Kim.

**Funding acquisition:** Hyun Kang.

**Investigation:** Hyun Kang, Geunjoo Choi.

**Methodology:** Beom Jin Kim.

**Project administration:** Beom Jin Kim.

**Software:** Hyun Kang.

**Supervision:** Beom Jin Kim, Jae Gyu Kim.

**Writing – original draft:** Hyun Kang.

**Writing – review & editing:** Beom Jin Kim, Jae Gyu Kim.

Beom Jin Kim orcid: 0000-0002-0938-6697
